# Exemestane increases the number and improves the morphology of cultured pre-antral follicles from transmasculine ovarian cortical tissue

**DOI:** 10.1016/j.isci.2026.116992

**Published:** 2026-08-11

**Authors:** Hui Cheng, Fu Wei, Tessa H.R. Stolk, Judith A. Huirne, Wouter van Vugt, Davy Cats, Hailiang Mei, Kevin A.J. Brewster, Elena Sánchez-López, Martin Giera, Gonneke S.K. Pilgram, Lucette A.J. van Der Westerlaken, Norah M. van Mello, Susana M. Chuva de Sousa Lopes

**Affiliations:** 1 Department of Anatomy and Embryology, Leiden University Medical Center, 2333 ZC Leiden, the Netherlands; 2 The Novo Nordisk Foundation Center for Stem Cell Medicine (reNEW), Leiden University Medical Center, 2333 ZC Leiden, the Netherlands; 3 Amsterdam UMC Location Vrije University Amsterdam, Department of Obstetrics and Gynecology, 1081 HV Amsterdam, the Netherlands; 4 Amsterdam UMC, Centre of Expertise on Gender Dysphoria, 1081 HV Amsterdam, the Netherlands; 5 Amsterdam Reproduction and Development Research Institute, 1081 HV Amsterdam, the Netherlands; 6 Sequencing Analysis Support Core, Leiden University Medical Center, 2333 ZC Leiden, the Netherlands; 7 Center for Proteomics and Metabolomics, Leiden University Medical Center, 2300 RC Leiden, the Netherlands; 8 Department of Gynecology, Leiden University Medical Center, 2333 ZA Leiden, the Netherlands; 9 Ghent-Fertility and Stem Cell Team (G-FAST), Department of Reproductive Medicine, Ghent University Hospital, 9000 Ghent, Belgium

**Keywords:** fertility preservation, follicular growth, culture, human, oocyte, granulosa cells, ovarian cortical tissue, steroidogenesis, luteinization, exemestane

## Abstract

To enhance fertility preservation options, it would be beneficial to develop culture platforms starting from cryopreserved-thawed ovarian cortical tissue. Importantly, ovarian cortical tissue from cisgender females (cOVA) and transmasculine individuals (tOVA) respond differently to culture and may require specific conditions to support optimal folliculogenesis. We explored the role of CYP19A1-inhibitor exemestane on the emergence of pre-antral follicles during culture to equalize the use of cOVA and tOVA. We compared follicular growth, morphology, and distribution between cOVA and tOVA using immunofluorescence and hormonal quantification. The inhibitor reduced the estrogen levels in both OVA types, while enhancing follicular morphology of tOVA. Moreover, treatment with exemestane decreased STAR expression in tOVA regulating progesterone locally. The addition of exemestane during this initial culture period may prove beneficial to obtain pre-antral follicles from tOVA more comparable to cOVA. These findings represent a step toward optimizing culture conditions to support early folliculogenesis.

## Highlights

• CYP19A1-inhibitor exemestane reduced luteinization in tOVA during culture
• Treatment with exemestane reduced production of estrogen in cOVA and tOVA
• Exemestane blocked STAR expression in tOVA during culture
• Exemestane improved the number of multilaminar follicles and oocyte diameter in tOVA

## Graphical abstract

**Figure:**
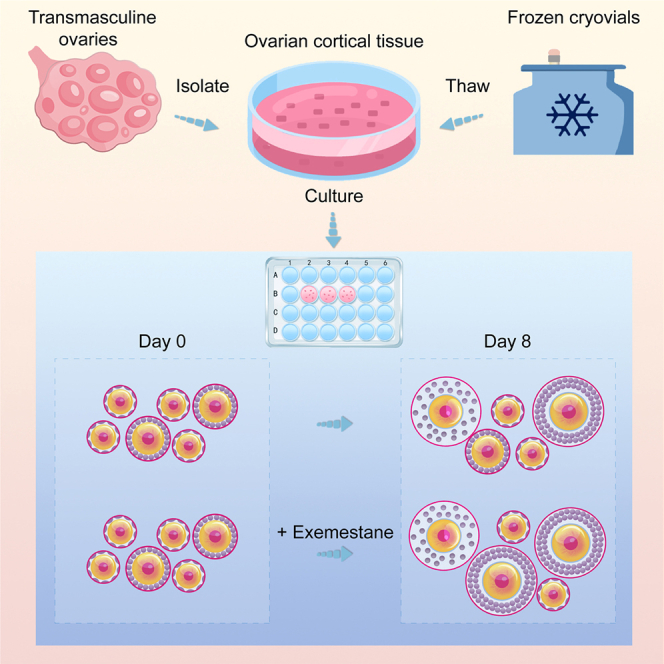

## Teaser

Reproductive medicine; Cell biology

## Introduction

Fertility preservation has become an essential aspect of reproductive medicine, particularly for individuals facing medical treatments or conditions that may impair their future reproductive potential. Advances in cancer therapies, including chemotherapy and radiation, while life-saving, can often lead to infertility due to their detrimental effects on the ovaries. Additionally, certain genetic conditions, autoimmune disorders, and gender-affirming treatments also pose risks to fertility.^1^ In response, various fertility preservation strategies have been developed to safeguard reproductive potential, enabling individuals to preserve fertility for future conception.^2^^,^^3^

Ovarian tissue cryopreservation (OTC) is one of the most promising fertility preservation techniques, particularly for individuals who cannot undergo standard methods, including egg or embryo freezing. OTC is an established clinical strategy capable of restoring endocrine function and achieving pregnancies,^4^^,^^5^ that involves the surgical removal and cryogenic freezing of ovarian cortical tissue, which contains the reserve of unilaminar follicles (primordial and primary follicles). Different methods for OCT are used, such as slow freezing and vitrification, but show differences regarding angiogenic potential, stromal integrity, VEGF secretion, and metabolic recovery of the tissue.^6^^,^^7^^,^^8^ The preserved tissue can be stored for extended periods, and when the patient is ready to conceive, the tissue can be thawed and transplanted back into the patient. Due to the risk of malignant cell contamination in the ovarian tissue, retransplantation is contraindicated in certain malignancies, such as epithelial ovarian cancer.^9^ Thus, establishing clinical protocols that effectively promote follicular growth and maturation *in vitro* would be highly beneficial, as it could lead to the development of functional oocytes for direct use in medically assisted reproduction. This approach has the potential to eliminate the need for ovarian tissue transplantation. In this regard, emerging strategies, such as *in vitro* maturation (after *in vivo* oocyte aspiration or after extraction from small antral follicles from ovarian tissue) and *in vitro* gametogenesis, that could capture the native ovarian environment as well as improve the metabolic and secretory recovery of thawed ovarian tissue, could provide complementary pathways to maximize fertility preservation options,^10^ further reducing the need for ovarian tissue transplantation.

Similar to cisgender individuals, many transmasculine individuals desire genetically related children^11^^,^^12^ and fertility preservation options for this population group are therefore important. Some transmasculine individuals choose for OTC at the time of hysterectomy and oophorectomy, after receiving gender affirming hormonal medications (typically testosterone) for long periods of time. Gender affirming hormonal medication may impact the gametic pool^13^ and their ability to generate embryos^14^; however, evidence from mice^15^ and humans^16^ indicate that oocyte potential for reproduction can be maintained despite testosterone exposure.

We have previously shown that *in vitro* culture of fresh and cryopreserved-thawed ovarian cortical tissue from transmasculine individuals (tOVA) does not result in the growth of high-quality pre-antral follicles when using culture protocols optimized for ovarian cortical tissue from cisgender females (cOVA),^17^ suggesting that culture adaptations may be necessary to effectively support early follicular development from tOVA. CYP19A1 is an aromatase that converts androgens (such as testosterone) into estrogen (such as estradiol) in granulosa cells (GCs) and estrogen is essential for follicular growth.^18^^,^^19^ Here, we investigated the effect of CYP19A1-inhibitor exemestane, an anti-breast cancer drug^20^ on the emergence of the pre-antral follicles after *in vitro* culture of freshly isolated and cryopreserved-thawed tOVA and cOVA. Our study suggested that the addition of exemestane lead to reduced luteinization in culture, including a reduction in the production of estrogen in both tOVA and cOVA, which resulted in comparable number and early follicular morphology at the pre-antral stage between the two groups.

## Results

### Pre-antral follicles from tOVA undergo premature luteinization in culture

**Figure 1:**
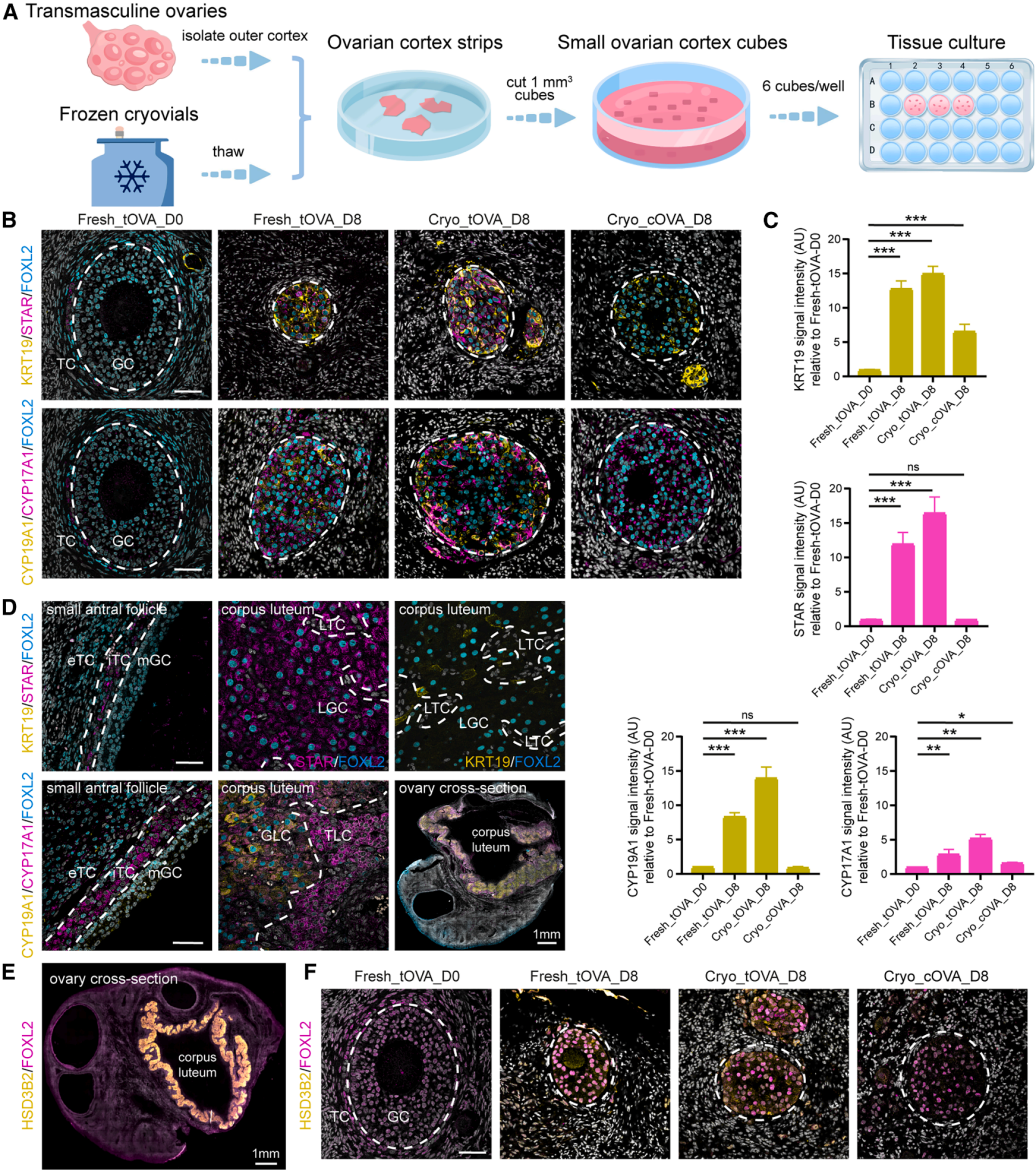
Pre-antral follicles from tOVA undergo premature luteinization in culture (A) Schematic workflow used to culture ovarian cortical tissue. (B) Immunofluorescence for KRT19, STAR, and FOXL2 (top row) and for CYP19A1, CYP17A1, and FOXL2 (bottom row) in multilaminar follicles from freshly isolated transmasculine ovarian cortical tissue (tOVA), cryopreserved-thawed tOVA, and cryopreserved-thawed cis female ovarian cortical tissue (cOVA) after isolation (day 0, D0) and following 8 days culture (D8). DAPI staining shows the nuclei in white; dashed lines demarcate the follicular basement membrane. GC, granulosa cells; TC, theca cells. Scale bars, 50 μm. (C) Quantification of KRT19, STAR, CYP19A1, and CYP17A1 signal intensities in multilaminar follicles using mean fluorescence intensity in arbitrary units (AU) relative to fresh_tOVA_D0. The results were presented as average ± standard deviation; the statistical test used was the Kruskal-Wallis test, followed by Dunn’s post hoc test for multiple comparisons (∗*p* < 0.05; ∗∗*p* < 0.01; ∗∗∗*p* < 0.001; ns, not significant *p* > 0.05). (D) Immunofluorescence for KRT19, STAR, and FOXL2 in the follicular wall of small antral follicles showing mural granulosa cells (mGC), internal theca cells (iTC), external theca cells (eTC); and corpus luteum showing lutein granulosa cells (LGC), and lutein theca cells (LTC). Dashed white lines show the borders between the different cell types of interest. Scale bars are 50 μm, except in the ovary cross-section (1 mm). (E) Immunofluorescence for HSD3B2 and FOXL2 in an ovary cross-section containing a corpus luteum. Scale bars, 1 mm. (F) Immunofluorescence for HSD3B2 and FOXL2 in multilaminar follicles from freshly isolated tOVA at D0 and D8, cryopreserved-thawed tOVA at D8, and cryopreserved-thawed cOVA at D8. DAPI staining shows the nuclei in white; dashed lines demarcate the follicular basement membrane. GC, granulosa cells; TC, theca cells. Scale bars, 50 μm. See also Table S1.

We cultured freshly isolated tOVA and cryopreserved-thawed tOVA and cOVA from different donors (Table S1) for 8 days (D8) using our published culture conditions (Figure 1A),^17^ that were based on the culture protocol from McLaughlin et al.^21^ After D8 culture, we observed that the FOXL2+ GCs from tOVA pre-antral (multilaminar) follicles (freshly isolated and cryopreserved-thawed) expressed significantly higher levels of KRT19, STAR, CYP17A1, and CYP19A1 compared to FOXL2+ GCs in tOVA before culture (day 0, D0) (Figures 1B and 1C). By contrast, FOXL2+ GCs present in D8 cultured cOVA expressed low levels of STAR and CYP19A1, comparable to GCs at D0 (Figures 1B and 1C).

The cellular identity of the human corpus luteum remains poorly described^22^; however, granulosa lutein cells express high levels of CYP19A1 (or P450arom) and theca lutein cells express high levels of CYP17A1 (or P450c17).^23^ We performed immunofluorescence on paraffin cross-sections of a (transmasculine) ovary containing a visible corpus luteum, as a third of transmasculine individuals on testosterone ovulate.^24^ We observed specific expression of STAR and CYP19A1 in FOXL2+ granulosa lutein cells in corpus luteum, in contrast to mural FOXL2+ GCs in small antral follicles that were both STAR and CYP19A1 negative (Figure 1D). Finally, we also used HSD3B2 (or 3β-HSD type 2), the main isoform of 3β-hydroxysteroid dehydrogenase expressed in the corpus luteum,^25^^,^^26^ as an additional marker associated with luteinization. The results confirmed high expression of HSD3B2 in the corpus luteum (Figure 1E). After D8 culture, FOXL2+ GCs in multilaminar follicles of tOVA showed moderate expression of HSD3B2 (Figure 1F). Together, this suggested that during the culture period the GCs of the multilaminar follicles of tOVA, and to some extent those of cOVA, became steroidogenic, undergoing some degree of luteinization prematurely, resulting in poor quality folliculogenesis.

### Treatment with exemestane reduced the production of estrogen in cOVA and tOVA

**Figure 2:**
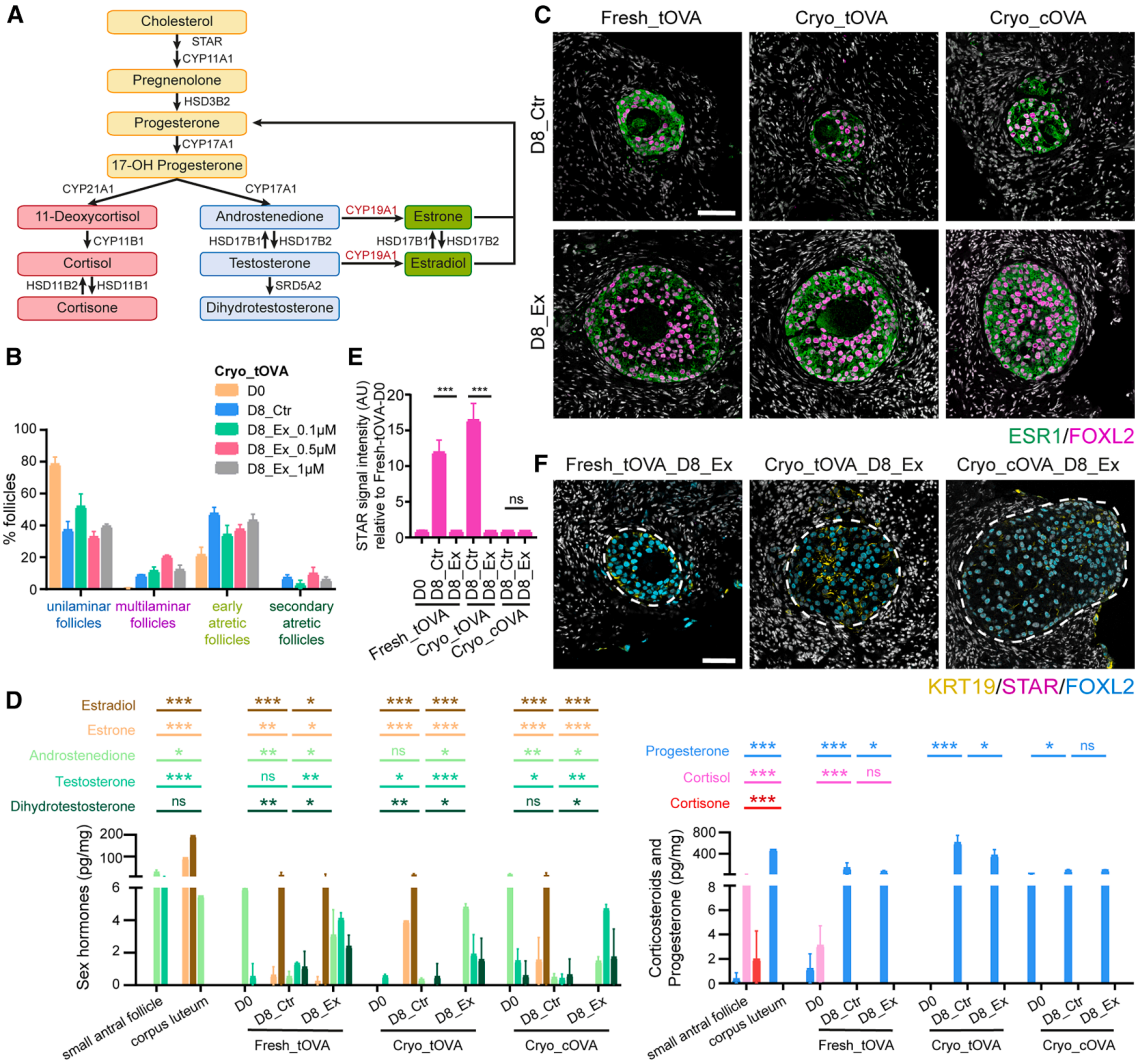
Treatment with exemestane reduced the production of estrogen and progesterone (A) Simplified diagram of steroid hormone synthesis pathway showing the respective enzymes. (B) Effect of different concentration of exemestane on the distribution of follicles (unilaminar, multilaminar, early atretic, and secondary atretic) present in cryopreserved-thawed transmasculine ovarian cortical tissue (tOVA) (*n* = 3 donors) after 8 days (D8) of culture compared to freshly isolated tOVA (D0) (*n* = 3 donors). The results shown are mean ± standard deviation from three independent experiments. (C) Immunofluorescence for ESR1 and FOXL2 in multilaminar follicles from freshly isolated tOVA, cryopreserved-thawed tOVA, and cryopreserved-thawed cis female ovarian cortical tissue (cOVA) after D8 culture in the absence (Ctr) of presence of 0.5 μM exemestane (Ex). DAPI staining shows the nuclei in white. Scale bars, 50 μm. (D) Expression levels of estradiol, estrone, androstenedione, testosterone, dihydrotestosterone, progesterone, cortisol and cortisone in fresh small antral follicles (*n* = 2 donors), corpus luteum (*n* = 1 donor), freshly isolated tOVA (*n* = 3 donors), cryo-thawed tOVA (*n* = 5 donors), and cryo-thawed cOVA (*n* = 5 donors) before (D0) and after culture (D8) in the absence (Ctr) of presence of 0.5 μM exemestane (Ex). The results were presented as average ± standard deviation; the statistical test used was the Kruskal-Wallis test, followed by Dunn’s post-hoc test for multiple comparisons (∗*p* < 0.05; ∗∗*p* < 0.01; ∗∗∗*p* < 0.001; ns, not significant *p* > 0.05). (E) Quantification of STAR signal intensity in multilaminar follicles using mean fluorescence intensity in arbitrary units (AU) relative to fresh_tOVA_D0. The results were presented as average ± standard deviation; the statistical test used was the Kruskal-Wallis test, followed by Dunn’s post-hoc test for multiple comparisons (∗*p* < 0.05; ∗∗*p* < 0.01; ∗∗∗*p* < 0.001; ns, not significant *p* > 0.05). (F) Immunofluorescence for KRT19, STAR, and FOXL2 in multilaminar follicles from freshly isolated tOVA, cryopreserved-thawed tOVA, and cryopreserved-thawed cOVA after D8 culture with 0.5 μM Ex. DAPI staining shows the nuclei in white; dashed lines demarcate the follicular basement membrane. GC, granulosa cells; TC, theca cells. Scale bars, 50 μm. See also Figure S1, Tables S1 and S2.

Luteinization leads to increased steroidogenesis (high levels of progesterone and estrogen)^27^; therefore, we tested whether counteracting the overexpression of CYP19A1 observed after D8 culture in tOVA, using the synthetic CYP19A1-inhibitor exemestane (6-methyleneandrosta-1,4-diene-3,17-dione), would influence the production of estrogen (estrone and estradiol) and progesterone in GCs (Figure 2A).

We first tested the effect of different concentrations of exemestane in ovarian follicles during culture and quantified the types of follicles based on morphology (unilaminar follicle, multilaminar follicle, early/unilaminar atretic, secondary/multilaminar atretic) (Figure S1A) as described previously.^17^ We selected 0.5 μM (0.15 μg/mL) exemestane for further analysis, as this concentration resulted in the highest percentage of multilaminar follicles after D8 culture in tOVA (Figure 2B). Next, we supplemented the culture medium with 0.5 μM exemestane and cultured freshly isolated tOVA and cryopreserved-thawed tOVA and cOVA for D8. We determined that FOXL2+ GCs of multilaminar follicles of cultured tOVA and cOVA expressed high levels of estrogen receptor alpha (ESR1) (Figure 2C) and low/absent levels of progesterone receptor (PGR) independently of treatment with exemestane (Figure S1B), with similar levels between the different groups of cultured cOVA and tOVA (Figure S1C).

We measured the effect of exemestane on the production of steroid hormones using liquid chromatography-tandem mass spectrometry (LC-MS/MS) (Table S2) on tissue extract of freshly isolated tOVA (*n* = 3 donors: tOVA132, tOVA133, tOVA134) and cryopreserved-thawed tOVA (*n* = 5 donors: tOVA100, tOVA107, tOVA108, tOVA136, tOVA137), and cOVA (*n* = 5 donors: cOVA12, cOVA22, cOVA66, cOVA76, cOVA84) (Table S1) before (D0) and after culture (D8). This was compared to the steroid production on tissue extract of freshly isolated small antral follicles (3× 2–5 mm diameter from *n* = 2 donors tOVA132 and tOVA134) and corpus luteum (1× tOVA135) (Figure 2D).

Regarding the levels of estrogen (estrone and estradiol), we observed an overall significant increase in the OVA tissue (cOVA and tOVA) after D8 culture compared to D0, although the levels remained much lower than those measured in corpus luteum, whereas the levels in exemestane-treated OVA remained low (Figure 2D). Conversely, the levels of androgens (androstenedione, testosterone, and dihydrotestosterone) increased in the exemestane-treated OVA tissue compared to the non-treated OVA tissue in both tOVA and cOVA (Figure 2D), suggesting that the treatment with exemestane successfully blocked androgen conversion to estrogen.

The levels of progesterone also increased significantly overall between D0 and D8 culture and showed a significant decrease in exemestane-treated in tOVA (freshly isolated and cryopreserved-thawed) (Figure 2D), whereas in cOVA the levels of progesterone remained comparable independently of exemestane treatment. Corticosteroids (cortisol and cortisone) were detected in the small antral follicles and freshly isolated tOVA, but not in the corpus luteum or cryopreserved OVA tissue. Independently of exemestane-treatment, corticosteroids were not detected in OVA tissue after D8 culture (Figure 2D). Our findings suggested that treatment with exemestane resulted in lower levels of estrogen in tOVA and cOVA tissue and lower levels of progesterone in tOVA tissue, therefore preventing this aspect of luteinization and contributing to equalize the quality of ovarian tissue in tOVA and cOVA after culture.

The reduction of progesterone levels in tOVA cultured in the presence of exemestane were intriguing due to the fact that the hypothalamic-pituitary-ovarian (HPO) axis is absent in the culture system, and therefore classical endocrine feedback mechanisms involving estrone/estradiol cannot directly regulate progesterone levels. However, the promoter region of *STAR* has a binding site for estrogen receptor^28^ and we observed high levels of STAR in tOVA after culture (Figures 1B and 1C). Interestingly, the expression of STAR in tOVA after treatment with exemestane remained low, similar to that tOVA at D0 (Figures 2E and 2F), and leading to the hypothesis that directs regulation of STAR in tOVA impacted production of progesterone.

### Exemestane increased the number of multilaminar follicles in tOVA after culture

**Figure 3:**
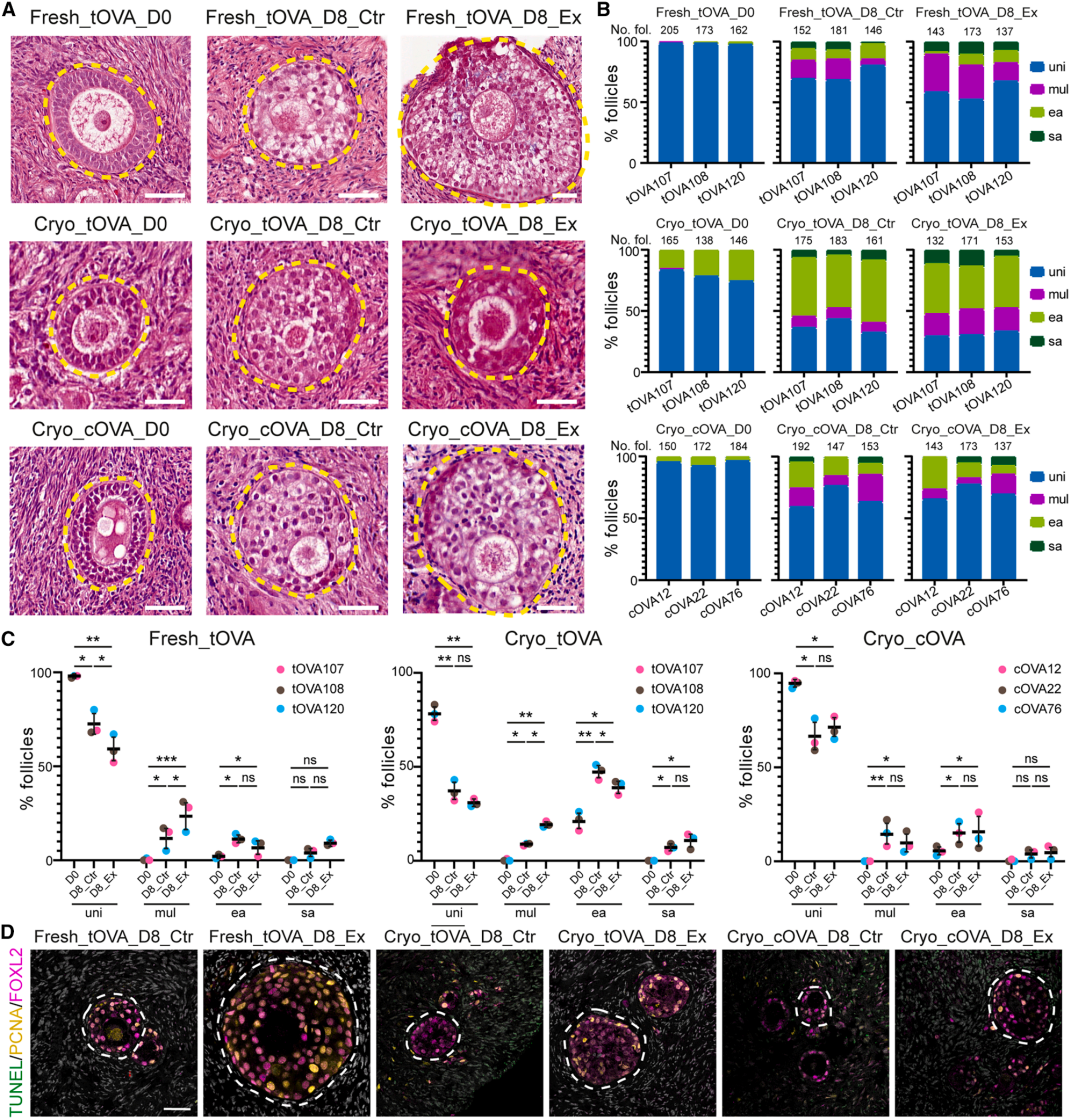
Exemestane improved the number of the multilaminar follicles in tOVA after culture (A) Hematoxylin and eosin staining showing multilaminar follicles in freshly isolated transmasculine ovarian cortical tissue (tOVA), cryopreserved-thawed tOVA and cryopreserved-thawed cis female ovarian cortical tissue (cOVA) before (D0) and after 8 days (D8) in the absence (Ctr) of presence of 0.5 μM exemestane (Ex). Dashed lines demarcate the follicular basement membrane. Scale bars, 50 μm. (B) Percentage of follicular distribution in freshly isolated tOVA, cryopreserved-thawed tOVA and cryopreserved-thawed cOVA per donor (*n* = 3 donors) at D0 and D8 in the absence (Ctr) of presence of 0.5 μM Ex. In the top of each bar is the total number of follicles counted per donor. (C) Percentage of follicular distribution in freshly isolated tOVA (*n* = 3 donors), cryopreserved-thawed tOVA (*n* = 3 donors), and cryopreserved-thawed cOVA (*n* = 3 donors) per follicular stage before and after culture. Black line is the average ± standard deviation. The statistical test used was the Kruskal-Wallis test, followed by Dunn’s post hoc test for multiple comparisons (∗*p* < 0.05; ∗∗*p* < 0.01; ∗∗∗*p* < 0.001; ns, not significant *p* > 0.05). (D) Immunofluorescence for PCNA and FOXL2 together with TUNEL staining in multilaminar follicles from freshly isolated tOVA, cryopreserved-thawed tOVA, and cryopreserved-thawed cOVA after D8 culture without (Ctr) or with 0.5 μM Ex. DAPI staining shows the nuclei in white; dashed lines demarcate the follicular basement membrane. Scale bars, 50 μm. uni, unilaminar follicle; mul, multilaminar follicle; ea, early atretic follicle; sa, secondary atretic follicle. See also Table S1.

To determine whether addition of exemestane with the resulting lower levels of estrogen and progesterone had a beneficial effect on the tOVA tissue, we quantified the number of follicles according to their diameter (unilaminar follicles <60 μm and multilaminar follicles >60 μm) (Figure 3A). We counted a total of 540 follicles in freshly isolated tOVA (*n* = 3 donors, average ± standard deviation [sd]: 180 ± 22) at D0 and from those the large majority were unilaminar follicles (average ± sd: 98% ± 1% unilaminar and 2% ± 1% atretic follicles) (Figures 3B and 3C). After D8 culture, we observed on average ± sd: 72% ± 8% unilaminar, 12% ± 7% multilaminar, 11% ± 2% early atretic, and 5% ± 3% secondary atretic follicles in the freshly isolated tOVA cultured without exemestane, whereas the freshly isolated tOVA from the same donors cultured with exemestane showed on average ± sd: 59% ± 8% unilaminar, 25% ± 8% multilaminar, 7% ± 4% early atretic, and 9% ± 3% secondary atretic follicles (Figure 3C). This suggested that treatment with exemestane resulted in a significant increase in the number of multilaminar follicles at D8 in freshly isolated tOVA, whereas the number of atretic follicles remained constant (Figure 3C).

We also investigated the effect of exemestane on follicular growth in cryopreserved-thawed tOVA of the same donors. We counted 449 follicles (*n* = 3 donors, average ± sd: 150 ± 14) at D0 (after thawing) and observed that on average (± sd) 22% ± 3% were already early atretic (Figures 3B and 3C), indicating that the cryopreservation and thawing procedure caused high levels of follicular atresia in tOVA. After D8 culture, the levels of atresia increased further in both tOVA cultured without exemestane (average ± sd: 37% ± 7% unilaminar, 9% ± 1% multilaminar, 47% ± 4% early atretic, and 7% ± 4% secondary atretic follicles in control group) and with exemestane (average ± sd: 31% ± 2% unilaminar, 19% ± 1% multilaminar, 39% ± 4% early atretic, and 11% ± 5% secondary atretic follicles) (Figure 3C). Most importantly, similar to freshly isolated tOVA, treating cryopreserved-thawed tOVA with exemestane also resulted in a significantly higher percentage of multilaminar follicles at D8 (Figures 3B and 3C).

Interestingly, exemestane treatment of cryopreserved-thawed cOVA (*n* = 3 donors) had no effect on the distribution of follicles observed at D8. The number of multilaminar follicles observed in cryopreserved-thawed cOVA (*n* = 3 donors) cultured D8 with exemestane (total follicles counted 453, with an average ± sd: 151 ± 19 follicles; average ± sd: 70% ± 6% unilaminar, 11% ± 6% multilaminar, 14% ± 7% early atretic, and 5% ± 4% secondary atretic follicles) or without exemestane (total follicles counted 492 with an average ± sd: 164 ± 24; average ± st: 66% ± 10% unilaminar, 14% ± 6% multilaminar, 16% ± 6% early atretic, and 4% ± 3% secondary atretic follicles) was not statistically different (Figures 3B and 3C). Moreover, the cryopreservation-thawing procedure seemed to have less impact on cOVA, than on tOVA at D0 and from the 506 follicles from cOVA (average ± sd: 169 ± 17) only an average ± sd: 6% ± 2% were early atretic follicles (Figures 3B and 3C).

The morphology of the multilaminar follicles after culture remained different from that of multilaminar follicles at D0, with some follicles showing lack of symmetry. We analyzed proliferation and apoptosis in non-atretic multilaminar follicles at D8 (Figure 3D) and observed high levels of proliferation marker PCNA and low/absent levels of TUNEL in FOXL2+ GCs of multilaminar follicles at D8, independently of treatment with exemestane. The stromal compartment showed low/absent levels of PCNA, but showed moderate levels of TUNEL in cryopreserved-thawed tOVA and cOVA (Figure 3D), indicating that cryopreservation by slow-freezing has a negative effect on the viability of the ovarian stromal compartment.

### Exemestane improved the diameter of oocytes in multilaminar follicles in tOVA after culture

**Figure 4:**
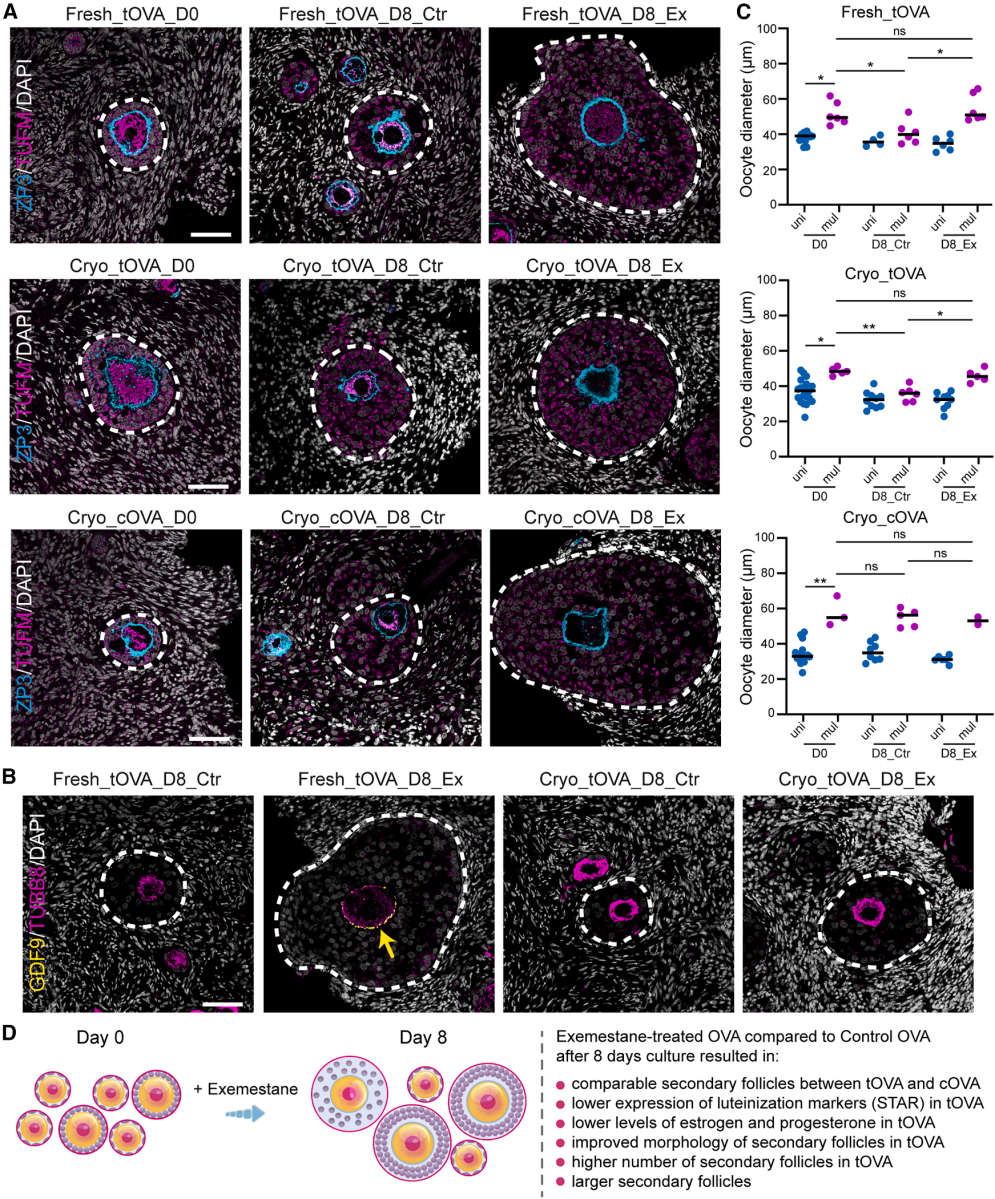
Exemestane improved the diameter of oocytes in multilaminar follicles in tOVA after culture (A) Immunofluorescence for ZP3 and TUFM in multilaminar follicles in freshly isolated transmasculine ovarian cortical tissue (tOVA), cryopreserved-thawed tOVA and cryopreserved-thawed cis female ovarian cortical tissue (cOVA) before (D0) and after 8 days (D8) in the absence (Ctr) of presence of 0.5 μM exemestane (Ex). Dashed lines demarcate the follicular basement membrane. Scale bars, 50 μm. (B) Immunofluorescence for GDF9 and TUBB8 in multilaminar follicles in freshly isolated tOVA and cryopreserved-thawed tOVA after D8 culture without (Ctr) or with 0.5 μM Ex. Dashed lines demarcate the follicular basement membrane. Yellow arrow points to GDF9 expression. Scale bars, 50 μm. (C) Oocyte diameter was measured in unilaminar and multilaminar follicles in freshly isolated tOVA cryopreserved-thawed tOVA, and cryopreserved-thawed cOVA at D0 and at D8 in the absence (Ctr) of presence of 0.5 μM Ex. Black line is the average. The statistical test used was the Kruskal-Wallis test, followed by Dunn’s post hoc test for multiple comparisons (∗*p* < 0.05; ∗∗*p* < 0.01; ∗∗∗*p* < 0.001; ns, not significant *p* > 0.05). (D) Overview summarizing the changes induced by exemestane in the proposed model. uni, unilaminar follicle; mul, multilaminar follicle.

To further investigate the effect exemestane during follicular growth, we performed immunofluorescence for the zona pellucida marker ZP3 and mitochondria marker TUFM, both markers were highly abundant in growing oocytes and growing GCs irrespectively of the culture group (Figure 4A). In addition, we also detected oocyte-specific TUBB8 and in large oocytes (>60 μm) GDF9 was upregulated (Figure 4B).

Next, we measured the diameter of the oocytes in both unilaminar and multilaminar follicles. We observed that the diameter of oocytes in the multilaminar follicles of cryopreserved-thawed cOVA before and after D8 culture (in the presence of absence of exemestane) were comparable (about 55 μm) (Figure 4C). Hence, treatment of cOVA with exemestane did not improve oocyte size after culture. However, treatment of tOVA (both freshly isolated and cryopreserved-thawed) with exemestane resulted in a significant increase of oocyte diameter, to a size comparable to oocytes in multilaminar follicles in tOVA at D0 (Figure 4C), suggesting a beneficial effect of exemestane in the culture of tOVA to obtain higher quality pre-antral follicles, comparable to those obtained in cOVA (Figure 4D).

## Discussion

The majority of transmasculine individuals express a desire to have biological children; however, uptake of fertility preservation interventions remains low.^11^^,^^12^^,^^16^ OTC is a valuable option for the transmasculine population, as it aims to preserve immature oocytes present in the ovarian cortex without the need to undergo ovarian stimulation.^2^ In tissue culture systems, the complete *in vitro* maturation of oocytes, from primary oocytes in unilaminar follicles to mature metaphase II oocytes, has been reported in two distinct multi-step culture protocols using freshly isolated ovarian tissue from cis gender adult females.^21^^,^^29^ However, the maturation of metaphase II oocytes starting from unilaminar follicles present in freshly isolated ovarian cortex from (adult) transmasculine individuals has not been reported and may require adaptations to the culture protocol used for cOVA.

Using the first step of the culture protocol developed by McLaughlin et al,^21^ we have previously reported the culture of cryopreserved-thawed tOVA and showed that tOVA were able to activate their GCs to develop into (pre-antral) multilaminar follicles.^17^ Here, we demonstrated that those GCs tend to undergo premature luteinization in culture, with consequences to the oocytes in the tissue that fail to increase in diameter during this transition. Note that we have not observed the presence of luteinized ovarian stroma in tOVA before culture, as others have described *in vivo*.^30^^,^^31^ Instead, we and others have reported that at least one-third of the transmasculine individuals undergo ovulation and their ovaries can contain bonafide corpus luteum and albicans.^24^^,^^31^ Moreover, the stromal luteinization may be explained by the presence of atretic follicles known to show aspects of luteinization.^32^

Interestingly, including 0.5 μM exemestane in the culture medium of tOVA for 8 days resulted in a higher number of (pre-antral) multilaminar follicles, oocytes with a larger diameter and a lower production of progesterone and estrogen, resulting in multilaminar follicles that were more comparable to cultured (pre-antral) multilaminar follicles from cOVA. Treatment with 0.5 μM exemestane in cOVA did not result in higher number of (pre-antral) multilaminar follicles, neither decreased production of progesterone. The CYP19A1-inhibitor exemestane has a direct role blocking the activity of CYP19A1,^33^ leading to a reduction in estrogen production. Interestingly, although the molecular mechanism remains largely unknown, treatment with exemestane has also been shown to reduce levels of progesterone in postmenopausal adult cis females with breast cancer, by reducing the levels of PGR.^34^ However, PGR was absent from (pre-antral) multilaminar follicles in tOVA and cOVA, before and after culture.

The effects of exemestane on the levels of progesterone in tOVA, but not on cOVA, were intriguing, since the culture system lacks the hypothalamic-pituitary-ovarian axis.^35^^,^^36^ However, after culture, the tOVA GCs showed pronounced levels of STAR, in contrast to cOVA GCs. Although this interpretation remains exploratory, we suggest that regulating the levels of estrogen during culture impacted STAR expression in tOVA GCs (which express estrogen receptors), and that subsequently modulated progesterone levels specifically in tOVA. The addition of CYP19A1-inhibitor exemestane during the first step of culture to promote follicular growth may help to equalize the use of tOVA and cOVA, by reducing STAR expression and lowering progesterone levels in tOVA to levels comparable to cOVA.

The present comparison between cryopreserved-thawed tOVA and cOVA provided a unique opportunity to assess early follicular growth (from dormant follicle to pre-antral follicle), using the first step of the multi-step culture system developed for freshly isolated cOVA by McLaughlin et al.^21^ Several key differences between tOVA and cOVA were observed. Firstly, from the donors analyzed the tOVA seemed more sensitive than cOVA to the slow freezing procedure resulting in higher percentage of atretic follicles, before and after culture. Secondly, tOVA showed pronounced STAR expression in GCs after culture, whereas cOVA did not, suggesting a differential regulation of cholesterol transport. Thirdly, while exemestane treatment reduced progesterone levels in tOVA, it had no significant effect on cOVA, indicating that tOVA may be more sensitive to CYP19A1 inhibition. These differences may be attributed to the long-term testosterone exposure in transmasculine individuals prior to OTC that could alter the expression of steroidogenic enzymes in the follicular niche.

In conclusion, based on the tOVA and cOVA analyzed, treatment with CYP19A1-inhibitor exemestane in tOVA seemed to equalize the number and morphology of (pre-antral) multilaminar follicles between tOVA and cOVA obtained after D8 culture, by reducing the hormones that induce premature luteinization in culture. The next step to promote folliculogenesis during culture of cOVA and tOVA will be to incorporate additional intraovarian factors, such as Activin A, follicle-stimulating hormone (FSH), and insulin growth factor (IGF) in the subsequent steps of the multi-step culture system to investigate the transition to the antral stage.^37^^,^^38^^,^^39^

### Limitations of the study

This study has several limitations, including a small sample size (*n* = 3 to *n* = 5), which may restrict the generalizability of findings to a broader population, underscoring the difficulty in obtaining ovarian material for scientific research. This aspect together with the high variability in follicular volume and distribution typical of the human ovarian tissue could undermine the power of the experimental approach and conclusions drawn. In addition, this study did not assess functional indicators of oocyte competence. Although LC-MS/MS provided valuable insights into steroid hormone production, the downstream effects on the next steps of follicular growth and oocyte maturation remain unexplored. The short 8-day culture duration does not fully capture long-term development. Hence, while the results are promising, additional research is needed to ensure the safety and efficiency of exposure to CYP19A1 inhibitors. Finally, as this study used ovarian cortical tissue from individuals exposed to long-term testosterone treatment, the applicability of this culture approach to additional clinical contexts remains to be investigated. Studies using ovarian tissue from broader clinical backgrounds will be essential to validate our findings.

## Resource availability

### Lead contact

Further information and requests for resources and reagents should be directed to Susana M. Chuva de Sousa Lopes (lopes@lumc.nl).

### Materials availability

This study did not generate new unique reagents.

### Data and code availability

• Data reported in this paper will be shared by the lead contact upon request.
• This paper does not report original code.
• Any additional information required to reanalyze the data reported in this paper is available from the lead contact upon request.

## Acknowledgments

We thank the patients who donated tissue for this study and Huan Cheng for drawing the schematic workflow. This research was funded by the 10.13039/501100000781European Research Council consolidator grant OVOGROWTH (ERC-CoG-2016-725722) to S.M.C.d.S.L., the 10.13039/501100009708Novo Nordisk Foundation (reNEW NNF21CC0073729) to H.C., F.W., K.A.J.B., E.S.-L., M.G., and S.M.C.d.S.L., and 10.13039/501100004543China Scholarship Council (CSC 202008320362 and CSC 202008450034) to H.C. and F.W., respectively.

## Author contributions

H.C. and S.M.C.d.S.L. conceived the study; H.C., F.W., T.H.R.S., J.A.H., W.v.V., G.S.K.P., L.A.J.v.D.W., N.M.v.M., and S.M.C.d.S.L. contributed to material collection; L.A.J.v.D.W., N.M.v.M., and S.M.C.d.S.L. arranged the ethical permits; H.C., F.W., D.C., H.M., K.A.J.B., E.S.-L., and M.G. generated data. All authors contributed to data analysis, manuscript writing and approved the submitted version. All authors provided the final approval of the version to be published.

## Declaration of interests

The authors declare no competing interests.

## STAR★Methods

### Key resources table

**Table:** 

REAGENT or RESOURCE	SOURCE	IDENTIFIER
**Biological samples**		

Ovarian tissue from transmasculine individuals	Amsterdam UMC, the Netherlands	NA
Ovarian tissue from cisgender females	LUMC, the Netherlands	NA

**Antibodies**		

Rabbit monoclonal anti-CYP17A1 Dilution 1:200	Abcam	Cat: ab125022 RRID: AB_10975095
Mouse monoclonal anti-CYP19A1 Dilution 1:200	Santa Cruz	Cat: sc-374176 RRID: AB_10986411
Rabbit monoclonal anti-KRT19 Dilution 1:200	Abcam	Cat: ab76539 RRID: AB_1523469
Mouse monoclonal anti-STAR Dilution 1:200	Santa Cruz	Cat: sc-166821 RRID: AB_2197661
Goat polyclonal anti-FOXL2 Dilution 1:250	Bio-Techne	Cat: NB100-1277 RRID: AB_2106188
Mouse monoclonal anti-TUFM Dilution 1:200	Bio-connect	Cat: AMAb90966 RRID: AB_2665738
Goat polyclonal anti-ZP3 Dilution 1:200	Bio-connect	Cat: sc-23715 RRID: AB_2220639
Rabbit monoclonal anti-PGR Dilution 1:200	Cell Signaling Technology	Cat: 8757 RRID: AB_2797144
Rabbit polyclonal anti-ESR1 Dilution 1:200	Invitrogen	Cat: PA1308 RRID: AB_325813
Mouse monoclonal anti-PCNA Dilution 1:200	Santa Cruz	Cat: sc-56 RRID: AB_ 628110
Rabbit polyclonal anti-TUBB8 Dilution 1:200	Abbexa	Cat: abx006140 RRID: NA
Mouse monoclonal anti-HSD3B2 Dilution 1:200	Santa Cruz	Cat: sc-515120 RRID: AB_2721058
Goat polyclonal anti-GDF9 Dilution 1:200	Santa Cruz	Cat: sc-12244 RRID: AB_2279069
Alexa Fluor 488 donkey anti-rabbit IgG Dilution 1:500	Life Technologies	Cat: A-21206 RRID: AB_2535792
Alexa Fluor 594 donkey anti-mouse IgG Dilution 1:500	Life Technologies	Cat: A-21203 RRID: AB_141633
Alexa Fluor 647 donkey anti-goat IgG Dilution 1:500	Life Technologies	Cat: A-21447 RRID: AB_141844
DAPI Dilution 1:500	Life Technologies	Cat: D1306

**Chemicals, peptides, and recombinant proteins**		

0.9% NaCl solution	Fresenius Kabi	Cat: B230551
Ethylene glycol	Merck	Cat: 1.00949.1000
Sucrose	Sigma-Aldrich	Cat: S0389-500G
Phosphate-buffered saline (PBS)	Thermo Fisher	Cat: 14190-094
Insulin-Transferrin-Selenium-Sodium Pyruvate (ITS-A) (100X)	Thermo Fisher Scientific	Cat: 51300044
GlutaMAX	Thermo Fisher	Cat: 35050-038
HEPES	Thermo Fisher	Cat: 15630-049
Penicillin-streptomycin	Life Technologies	Cat: 15070063
Ascorbic acid	Merck	Cat: A92902
Paraformaldehyde (PFA)	Merck	Cat: 1.04005.1000
Trizma base	Merck	Cat: T6066
EDTA	VWR	Cat: M101
Tween-20	Merck	Cat: 822184
Cortisol	Sigma-Aldrich	Cat: H4001
Cortisone	Sigma-Aldrich	Cat: C2755
Testosterone	Sigma-Aldrich	Cat: 86500
Dihydrotestosterone	Sigma-Aldrich	Cat: 10300
Androstenedione	Phytolab	Cat: PHL89562
Estrone	Supelco	Cat: PHR1535
Progesterone	Sigma-Aldrich	Cat: 46665
β-estradiol	Sigma-Aldrich	Cat: E2758
Cortisol-d4	Sigma-Aldrich	Cat: C113
Testosterone-d3	Sigma-Aldrich	Cat: T046
Progesterone-d9	Sigma-Aldrich	Cat: P070
Bovine serum albumin (BSA)	Merck	Cat: 10735086001
Human serum albumin (HSA)	CSL Behring	Cat: RVG105901
McCoy’s 5A medium	Invitrogen	Cat: 26600-023
Recombinant human follicle-stimulating hormone (rhFSH)	Merck	Cat: GONAL-F 450U/0.75ML

**Other**		

Scalpels	Swann-Morton	Cat: 0507
Bead bath	Lab Armor	Cat: 74300-714
24-well plates	TPP	Cat: 92024
Shandon Excelsior ES tissue processor	Thermo Fisher Scientific	Cat: A78410120
Microtome	Leica Microsystems	Cat: RM2065
StarFrost microscope slides	Waldemar Knittel	Cat: 3057-1
TissueWave 2 Microwave	Thermo Fisher Scientific	Cat: B35600002
Panoramic 250 digital scanner	Budapest	Cat: 3DHISTECH Ltd
SP8 TC inverted confocal microscope	Leica	NA
Bullet Blender™ 24	Next Advance Inc	NA
C18 SPE cartridges	Waters, MA	Cat: WAT054945
HPLC system model	Shimadzu	Cat: SIL-40
Sciex QTrap 6500+ mass spectrometer	Sciex	NA
C18 pre-column	Phenomenex	Cat: AJ0-8782
*In Situ* Cell Death Detection Kit	Roche	Cat: 11684795910

**Software and algorithms**		

GraphPad Prism v9	GraphPad Software Inc	https://www.graphpad.com
ImageJ v1.8	Schindelin et al.^40^	https://imagej.net/ij/features.html
CaseViewer v2.4.0	3DHISTECH	https://www.3dhistech.com
LAS X v3.7.4	Leica	https://www.leica-microsystems.com/products/microscope-software/p/leica-las-x-ls/

### Experimental model and study participant details

#### Ethical approval for human tissue collection

This study was conducted in accordance with the guidelines of the Declaration of Helsinki, with a letter of non-objection from the Medical Ethical Committee of the Leiden University Medical Center (B16.050, B18.029, B22.3077). Informed consent was obtained from all donors.

#### Experimental models/study participants

Information on age and gender of the donors (all assigned female at birth) of human ovarian tissue used in this study is presented in Table S1. Information regarding ancestry, race, and ethnicity was not available. We acknowledge limitations regarding association with gender and age due to the small sample size.

One group of donors were individuals who received testosterone treatment for more than one year and were undergoing gender-affirming surgery (transmasculine donors; *n* = 11) at the Amsterdam UMC hospital. In that case, the whole ovaries were donated for research. The other group of donors (cis female donors; *n* = 5) underwent oophorectomy for fertility preservation before chemotherapy at the Leiden University Medical Center and provided prior written consent for the use of their cryopreserved ovarian tissue for scientific research in the event of their death.

### Method details

#### Ovarian cortex preparation, cryopreservation and thawing

Adult ovaries from cis female donors were cut in half and washed in a 0.9% NaCl solution. The ovarian cortex (1 mm thick) was prepared for cryopreservation as previously described.^17^ Briefly, one or two cortical strips were placed in cryovials containing 1 ml of cryoprotectant solution (0.1 M sucrose and 1.5 M ethylene glycol in phosphate-buffered saline [PBS]). The tissue was equilibrated in the solution for 30 minutes before undergoing slow freezing using a Planer Kryosave Compact Controlled Rate Freezer System. The freezing protocol involved sequential cooling, manual seeding at -9°C, and rapid cooling to -140°C, followed by storage in liquid nitrogen (-196°C).

Thawing was performed by submerging cryovials in a 37°C bead bath until the ovarian strips were fully thawed (3-5 minutes). To remove the cryoprotectant, the strips were sequentially washed in a solution of 0.75 M ethylene glycol and 0.25 M sucrose in PBS at room temperature (RT) with intermittent shaking for 10 minutes (min). This was followed by 10 min in a solution of 0.35 M sucrose in PBS, followed by 10 min in PBS.

The ovaries of transmasculine donors were either processed identically to that of cis female donors used for OTC and used freshly-isolated or cryopreserved-thawed; or fixed for immunofluorescence or frozen for hormonal quantification. Two transmasculine donors showed one ovary containing one corpus luteum. In that case, half of the ovary was fixed for histology and immunofluorescence. In addition, from those ovaries several small antral follicles and a small piece of corpus luteum were individually collected in 1.5ml tubes and frozen at -80C for further use for hormonal quantification.

#### Culture of ovarian cortical tissue

Freshly-isolated and cryopreserved-thawed ovarian cortex strips were cultured essentially as previously described.^17^^,^^21^ The strips were manually cut into 1 mm^3^ cubes using scalpels and the cubes were then cultured in 24-well plates (5-8 cubes per well) at 37°C in 5% CO_2_ in a humidified incubator for 8 days. Each well contained 1 ml of culture medium (McCoy’s 5A medium supplemented with 1 ng/ml rhFSH, 0.1% HSA, 1% ITS-A, 3 mM glutaMAX, 20 mM HEPES, 0.1% penicillin-streptomycin, and 50 μg/ml ascorbic acid) with or without exemestane (0.1 μM, 0.5 μM, 1 μM). Every other day halve of the culture medium was replaced.

#### Histochemistry and immunofluorescence

Freshly-isolated and cultured ovarian tissue cubes were fixed in 4% PFA overnight at 4°C and used for immunofluorescence or histochemistry [hematoxylin and eosin (HE) staining] as previously described.^17^ Briefly, after fixation the ovarian cortical tissue cubes were embedded in paraffin, and serially sectioned at a thickness of 5 μm. Eight sections per cube (5-8 cubes per condition per donor), with a separation of 40 μm between sections to avoid double-counting the same follicle, were used for HE and further quantification.

For immunofluorescence staining, Tris-EDTA buffer (10 mM Trizma base, 1 mM EDTA, pH 9.0) was used for antigen retrieval. The primary antibodies used were goat anti-FOXL2, goat anti-ZP3, mouse anti-PCNA, goat anti-GDF9, rabbit anti-TUBB8, mouse anti-STAR, mouse anti-HSD3B2, mouse anti-CYP19A1, mouse anti-TUFM, rabbit anti-CYP17A1, rabbit anti-KRT19, rabbit anti-PGR and rabbit anti-ESR1. The secondary antibodies used were Alexa Fluor 488 donkey anti-rabbit IgG, Alexa Fluor 594 donkey anti-mouse IgG, and Alexa Fluor 647 donkey anti-goat IgG. The TUNEL assay was performed using the *In Situ* Cell Death Detection kit following the manufacturer’s instructions.

#### Liquid chromatography-tandem mass spectrometry (LC-MS) analysis

Frozen tOVA, cOVA, isolated small antral follicles and pieces of corpus luteum were homogenized in chilled LC-MS-grade water to achieve a final concentration of 100 mg/ml in tissue extract. To 50 μl of tissue homogenate, 600 μl ice-cold LC-MS grade methanol, 150 μl water and 15 μl of the internal standard (IS) mix (100 ng/ml of cortisol-d4, testosterone-d3 and progesterone-d9 in methanol) were added. Samples were briefly vortexed, kept at -20°C for 20 min and centrifuged at 18000x*g* for 10 min at 4°C. The supernatants were transferred to 5 ml tubes containing 2 ml of water and 2 μl of formic acid. Sep-Pak 3 ml C18 solid phase extraction cartridges (Waters, MA, USA) were used for sample preparation. Briefly, SPE cartridges were conditioned with methanol and water before samples were applied. Cartridges were washed with water and n-hexane. Samples were then eluted with 3 ml methyl formate into glass vials. Samples were dried under a gentle stream of liquid nitrogen in a water bath kept at 40°C and then stored at -80°C. On the day of analysis, samples were reconstituted in 60 μl LC-MS grade methanol, vortexed for 10 seconds and sonicated for 1 min. Next, 90 μl of LC-MS grade water was added and samples were vortexed and transferred into micro-vial inserts and placed in the autosampler for LC-MS/MS analysis. Monitored multiple reaction monitoring (MRM) transitions used for steroid quantification (Table S2). All other LC-MS/MS parameters were identical to those described earlier,^41^ except the curtain gas, which was set to 30 psi.

### Quantification and statistical analysis

#### Imaging and quantification criteria for follicles and oocytes

Imaging of HE-stained slides was performed using a Panoramic 250 digital scanner with CaseViewer software. Follicle quantification was performed on 8 paraffin sections (5 μm thickness) separated by 40 μm per cube (5–8 ovarian cubes per condition per donor) and measurement of oocyte diameter on those sections were performed as previously described.^17^ We classified follicular stages (unilaminar, multilaminar, early atretic and secondary atretic) as previously.^17^^,^^42^ Only follicles containing a clearly visible oocyte nucleus were counted to ensure accuracy and avoid double-counting.

The total number of follicles present in all 8 sections per cube per donor per condition were summed (total follicle count per donor per condition), and the percentage of follicular stage per donor per condition was calculated relative to the total number of follicles counted per donor per condition.

For immunofluorescence, images were captured using an SP8 TC inverted confocal microscope equipped with LAS software. Mean fluorescence intensity (MFI) on the images was calculated using ImageJ/Fiji.^40^ For each channel, a threshold was applied to isolate the specific fluorescent signal from background noise. Following threshold selection, the measurement parameters were defined (Analyze → Set Measurements), with “Mean gray value” and “Limit to threshold” enabled. MFI was defined as Integrated Density (IntDen)/Area.

#### Steroid quantification

For steroid quantification, a calibration line was prepared using authentic standards at concentrations ranging from 0.009 to 160 ng/ml (12 levels) for all steroids except β-estradiol, which ranged from 0.45 to 500 ng/ml (8 levels). Each solution included 15 μl of the IS mix (100 ng/ml in methanol), yielding a final composition of 40:60 (v/v) methanol:water and a total volume of 150 μl. Steroid concentrations are reported as pg/g, normalized to the initial tissue weight.

#### Statistical analysis

Data were analyzed using GraphPad Prism v9.0.1. Statistical significance was determined using the Kruskal-Wallis test, a non-parametric alternative to one-way ANOVA, followed by Dunn’s post-hoc test for multiple comparisons for steroid quantification, follicular quantification, fluorescence intensity analysis and oocyte diameter analysis. Statistical significance was considered with *p*-value < 0.05 (∗), < 0.01 (∗∗) and < 0.001 (∗∗∗).
