# Supplementary material for: Exemestane increases the number and improves the morphology of cultured pre-antral follicles from transmasculine ovarian cortical tissue
Source: iScience. 2026 Aug 11;29(8):116992. doi: 10.1016/j.isci.2026.116992 (PMC13486770; doi:10.1016/j.isci.2026.116992)
Supplement: Supplementary file 1 [file mmc1.pdf]

iScience, Volume *29*

Supplemental information

Exemestane increases the number and improves the morphology of cultured pre-antral follicles from transmasculine ovarian cortical tissue

Hui Cheng, Fu Wei, Tessa H.R. Stolk, Judith A. Huirne, Wouter van Vugt, Davy Cats, Hailiang Mei, Kevin A.J. Brewster, Elena Sánchez-López, Martin Giera, Gonneke S.K. Pilgram, Lucette A.J. van Der Westerlaken, Norah M. van Mello, and Susana M. Chuva de Sousa Lopes

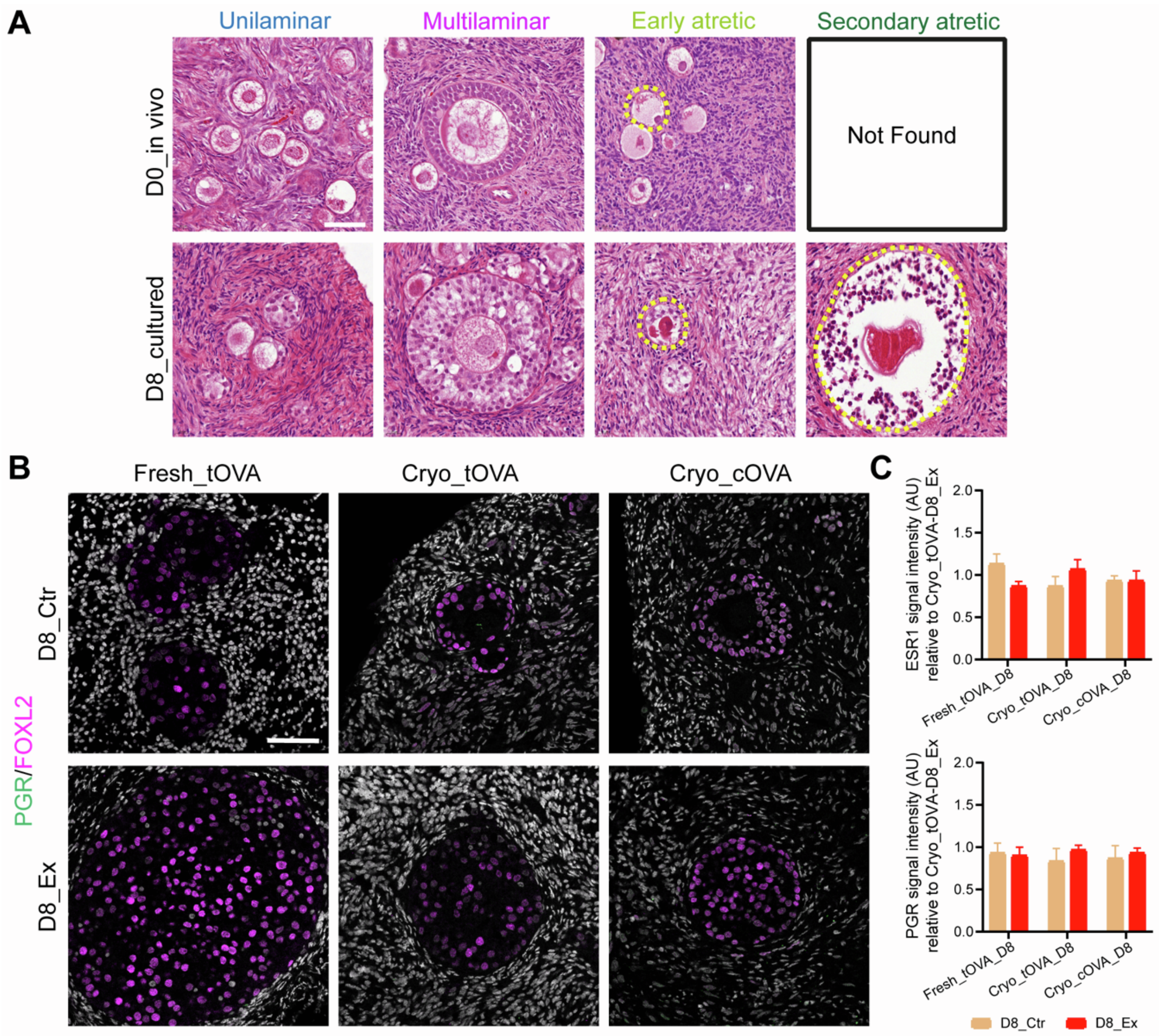

Figure S1. Follicular types and expression of PGR/ESR1 in OVA.
(A) Hematoxylin and Eosin staining showing different follicular types in vivo and after culture (day 8, D8). Note that we were unable to find atretic secondary follicles in vivo. Yellow dashed line demarcates the follicular basement membrane in atretic follicles. Scale bar is 50 µm.
(B) Immunofluorescence for PGR and FOXL2 in multilaminar follicles from freshly-isolated transmasculine ovarian cortical tissue (tOVA), cryopreserved-thawed tOVA, and cryopreserved-thawed cis female ovarian cortical tissue (cOVA) after D8 culture in the absence (Ctr) of presence of 0,5 mM Exemestane (Ex). DAPI staining shows the nuclei in white. Scale bar is 50 µm.
(C) Quantification of ESR1 and PGR signal intensities in multilaminar follicles using mean fluorescence intensity in arbitrary units (AU) relative to cryo_tOVA-D8_Ex. The results were presented as mean ± standard deviation; the statistical test used was the one-way ANOVA followed by Tukey's multiple comparison test, but none of the comparisons were significant ($p > 0.05$).
See also **Figure 2C**.

Table S1. Characteristics of the donors of ovarian cortical tissue.

Donor ID[a]	Age (years)	Type of gender affirming hormone therapy	Duration of gender affirming hormone therapy (months)	Period of cryopreservation (years)
tOVA43	20	Nebido	30	–
tOVA100	26	Nebido	20	–
tOVA107	19	Nebido	26	–
tOVA108	19	Androgel	38	–
tOVA120	18	Sustanon	41	–
tOVA132	24	Sustanon	24	–
tOVA133	24	Sustanon	47	–
tOVA134	20	Sustanon	60	–
tOVA135	21	Nebido	23	–
tOVA136	33	Nebido	19	–
tOVA137	36	Sustanon	70	–
cOVA12	34	–	–	17
cOVA22	31	–	–	16
cOVA66	17	–	–	7
cOVA76	17	–	–	9
cOVA84	17	–	–	7

[a]tOVA, ovarian tissue from transmasculine donor; cOVA, ovarian tissue from cis female donor

Table S2. MRM transitions used for the steroid quantification and the respective deuterated internal standards (ISs).

Steroid	IS used for correction	Retention time (min)	Nominal mass (Da)	$[M+H]^+$ (*m/z*)	Experimental MRM transition (Q1 → Q3, *m/z*)	Declustering potential (V)	Collision energy (V)	Collision cell exit potential (V)
Cortisone	Cortisol-d_4	2.2	360	361.2	361.1 → 163.1	111	31	10
Cortisol	Cortisol-d_4	2.5	362	363.2	363.3 → 121.1	66	31	14
Androstendione	Testosterone-d_3	5.1	286	287.1	287.1 → 97.0	66	29	10
Estrone	Testosterone-d_3	5.2	270	271.1	271.1 → 159.1	31	29	14
β-estradiol	Testosterone-d_3	5.2	272	273.1	273.1 → 107.0	66	43	18
Testosterone	Testosterone-d_3	5.3	288	289.2	289.1 → 97.0	81	27	10
Dihydrotestosterone	Testosterone-d_3	5.9	290	291.2	291.1 → 255.2	61	23	14
Progesterone	Progesterone-d_9	6.2	314	315.2	315.1 → 109.0	66	31	10
Cortisol-d_4 (IS)	-	2.5	366	367.2	367.1 → 121.1	186	31	14
Testosterone-d_3 (IS)	-	5.3	291	292.2	292.1 → 97.0	106	29	10
Progesterone-d_9 (IS)	-	6.2	323	324.2	324.1 → 100.0	81	29	12

IS, internal standard
